# Thiazolidinediones play a positive role in the vascular endothelium and inhibit plaque progression in diabetic patients with coronary atherosclerosis: A systematic review and meta-analysis

**DOI:** 10.3389/fcvm.2022.1043406

**Published:** 2022-11-29

**Authors:** Cheng Yuan Xue, Meng Qi Zhou, Qi Yan Zheng, Jin Hui Zhang, Wei Ting Cheng, Xue Hui Bai, Fen Zhou, Ai Ming Wu, Bo Nie, Wei Jing Liu, Li Xia Lou

**Affiliations:** ^1^Dongzhimen Hospital, Beijing University of Chinese Medicine, Beijing, China; ^2^Key Laboratory of Chinese Internal Medicine of Ministry of Education and Beijing, Beijing, China; ^3^Shenzhen Traditional Chinese Medicine Hospital, Shenzhen, China; ^4^Nursing School, Beijing University of Chinese Medicine, Beijing, China

**Keywords:** thiazolidinediones (TZDs), rosiglitazone, pioglitazone, endothelium, plaque, diabetes, coronary atherosclerosis

## Abstract

Rosiglitazone (Avandia) and pioglitazone (Actos) belong to the class of thiazolidinediones (TZDs) drugs that act by increasing insulin sensitivity and are widely used for treating diabetic patients with insulin resistance. TZDs exhibit anti-inflammatory and antioxidant properties, then may play an active role in inhibiting plaque formation and coronary atherosclerosis. But the results of evidence-based medicine suggest that TZDs may increase the risk of cardiovascular adverse events. To explore the dispute in depth, our meta-analysis aimed to evaluate the changes in vascular endothelial and plaque-related indicators following treatment with TZDs in diabetic patients with coronary atherosclerosis. According to our meta-analysis, TZDs showed an inhibiting effect on plaque progression and a protective effect on the vascular endothelium in patients with diabetes and coronary atherosclerosis. Interestingly, these effects may not depend on the regulation of inflammation and lipid metabolism. By this token, TZDs may develop a potential protective effect on myocardial infarction.

**Systematic review registration:**

[https://www.crd.york.ac.uk/prospero/], identifier [CRD42021231663].

## Introduction

Globally, 537 million adults (20–79 years) are living with diabetes. This number is predicted to rise to 643 million by 2030 and 784 million by 2045 (1). Diabetes has been considered to be associated with an increased risk of cardiovascular disease (2). Many studies have shown that people with diabetes are 2-4 times more likely than the general population to develop cardiovascular disease and have an increased mortality risk (3). There is an increasing number of patients who suffer from diabetes concomitant with cardiovascular disease. In general, simple diabetes and diabetes combined with cardiovascular disease have different treatment purposes and specific principles. Finding a drug with both hypoglycemic action and cardiovascular protecting effects is very necessary.

Rosiglitazone and pioglitazone belong to the class of thiazolidinediones (TZDs) that act by increasing insulin sensitivity and are widely used for treating patients with type 2 diabetes mellitus (T2DM). TZDs have been reported to have both anti-inflammatory and antioxidant functions, which are thought to be the major causes of coronary atherosclerosis (4). Accordingly, TZDs are believed to have a role in preventing coronary atherosclerotic heart disease. But currently, there has been controversy over TZDs in the cardiovascular field. A meta-analysis in May 2007 reported for the first time that rosiglitazone increased the risk of myocardial infarction by 43%, which showed that TZDs may have cardiovascular adverse effects (5). Subsequently, a series of evidence-based medical evidence was published that the increased risk of heart failure due to TZDs is relatively clear, but researchers failed to reach consistent conclusions on the myocardial infarction risk of TZDs. Recently, a meta-analysis with a sample size of 21156 summarized the effects of rosiglitazone on cardiovascular risk and mortality (6). The results suggested that rosiglitazone was associated with an increased cardiovascular risk, especially for heart failure events, the odds ratios for myocardial infarction were 1.17. But the authors were cautious about the result, because of the small confidence intervals and large diversity between different analysis methods. An umbrella review concludes: the risk ratios for heart failure between the pioglitazone intervention group and the control group were 1.40, which suggests a 40% increase in myocardial infarction risk after pioglitazone intervention (7). However, pioglitazone is well-known before to have a protective effect on cardiovascular disease including heart failure events in patients with diabetes (8). Hence, the relationship between TZDs and cardiovascular risk was still unclarified.

Endothelium, whose dysfunction is implicated in several pathophysiologic processes, including atheromatous plaque formation and coronary artery pathological changes, is considered to be an important target for TZDs in coronary atherosclerosis (9). The analysis and judgment of the effects of TZDs on endothelial-related indicators or atheromatous plaques will be of great help to our understanding of the association between TZDs and cardiovascular diseases, especially myocardial infarction. Our meta-analysis was designed to assess changes in vascular endothelial and plaque-related indices after rosiglitazone or pioglitazone treatment in patients with diabetes combined with coronary atherosclerosis, and to explore potential targets for the protective effects of TZDs in myocardial infarction.

## Materials and methods

This meta-analysis was reported following the Preferred Reporting Items for Systematic Reviews and Meta-Analyses guideline (PRISMA) (10), with our protocol registered in PROPERO as CRD42021231663.

### Literature search strategy

To find relevant peer-reviewed studies regarding TZDs and endothelium in diabetic patients with coronary atherosclerosis, different electronic databases including PubMed/Medline, EMBASE, Web of Science, and Cochrane Library were used. The search terms included “Coronary Artery Disease,” “Endothelium,” “TZDs,” “Glitazones,” “Rosiglitazone,” etc. The combinations of different search terms were used for identifying the relevant articles, and the search strategies were customized to suit each database (see Supplementary Table 1).

### Criteria for inclusion and exclusion

Inclusion criteria for this study were the following: (a) the study must be a randomized controlled trial design; (b) it should be published in a peer-reviewed journal in the English language; (c) studies should have clear diagnostic criteria about diabetic patients with coronary atherosclerosis; (type 2 diabetes mellitus according to the diagnostic criteria of T2DM published in the American Diabetes Association, and coronary heart disease or vascular stent surgery suggesting coronary atherosclerosis) (d) reported studies should be available in full text (not editorial, commentary, or abstract for conferences). Studies were excluded if (a) they were published in other languages than English and contained only qualitative data; (b) the intervention and control groups mixed up patients with other diseases; (c) the combination application of TZDs was present in the intervention group.

### Data extraction and management

Two authors (Cheng Yuan Xue and Meng Qi Zhou) reviewed the titles, abstracts, and/or full texts for each of the articles identified by the literature search after the removal of duplicates, aiming to determine the eligibility for this meta-analysis. During the study selection process, discrepancies were resolved by discussion with a third author (Qi Yan Zheng). All authors independently performed data extraction using standard extraction spreadsheets from the selected articles based on the inclusion criteria and enlisted them in a table. The following items were extracted from each study: author’s name (first author), year of publication, country, groups, gender distribution, mean age (years), several participants (male vs. female), vessel volume, lumen volume, plaque volume, neointima volume, flow-mediated dilatation, adiponectin, endothelin, NO, NOS, CRP, interleukin-6, TNF-α, triglycerides, total cholesterol, low-density lipoprotein cholesterol, high-density lipoprotein cholesterol. After the extraction of the data, the authors cross-checked the data tables and resolved any conflicts and inconsistencies during the data extraction process through discussion with each other.

### Quality assessment

The quality assessment of all included studies was conducted by using the Cochrane Collaboration “Risk of Bias” tool (11). We expand our analysis through the following six items: selection bias, performance bias, detection bias, attrition bias, reporting bias, and other biases. Each entry was also classified into three levels of bias risk: “high risk,” “low risk” and “uncertain risk”. The two evaluators evaluated the literature quality, respectively, and the differences were determined by a third party.

### Published bias

The number of studies on the same outcome measure was too small, so publication bias was tested without funnel plots.

### Statistical analysis

We used the statistical software named Review Manager V5.4.1 (Cochrane Collaboration, Copenhagen, Denmark) for the meta-analysis.^1^ Most of the results were analyzed using the fixed-effects model and forest plots. For strong heterogeneous results, we used the random-effect model as the statistical method, which seems to provide more conservative results than the fixed-effects model (12).

We calculated the standardized mean difference (SMD) with a corresponding 95% confidence interval (CI) for each parameter using the random-effects model. A *P*-value < 0.05 was considered a statistically significant difference between groups. The existence of heterogeneity among studies was evaluated using *I*^2^ and its resultant *P*-value using chi-squared tests. *I*^2^ values of 0–25%, 25–50%, 50–75% and above 75% correspondence to the heterogeneity as absence or very low, low, medium, and high. respectively, if the *P*-value was smaller than 0.1, the results were considered to be heterogeneous.

## Results

### Search results

**FIGURE 1:**
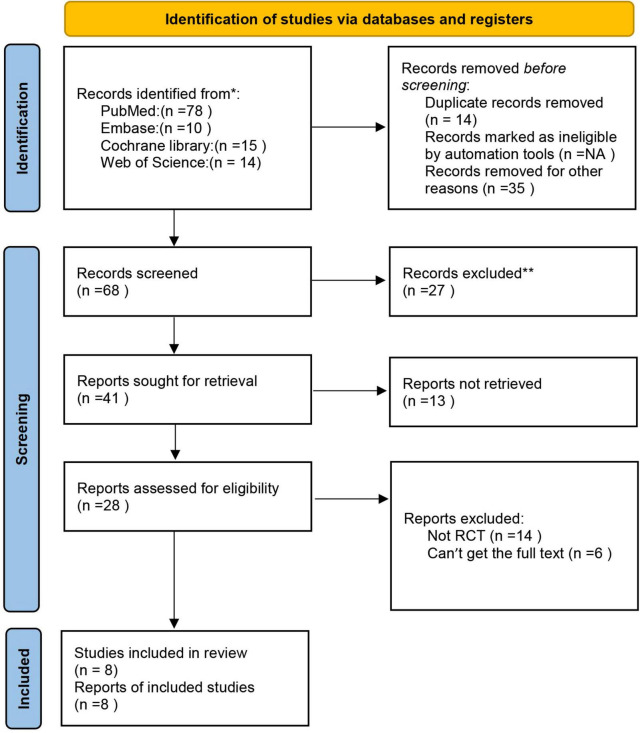
The flow diagram of the literature research and study selection according to the PRISMA guidelines.

As shown in Figure 1, a total of 117 studies were initially identified through different database searching. After the removal of duplication, additional screening, and analysis of the titles, abstracts, or full texts for each of the articles, 28 articles were included as eligible for this study. For articles where the full text is available, we read the full text carefully, 20 articles were excluded because they are not RCT, single-handed intervention or we can’t find the full text. Finally, 8 studies were included in the qualitative and quantitative review and meta-analysis of our study.

### Characteristics of included studies

The main characteristics of participants and interventions are presented in Table 1. There were 451 participants enrolled in the 8 studies.

**TABLE 1:** The included literature feature.

Study ID	Age/Year		Sample size		I	C	Duration
	I	C	I	C			
Marjorie Bastien et al. 2019 (13)	63.3 ± 7.4	64.8 ± 7.0	53	51	rosiglitazone	Placebo treatment	12 months
Olivier et al. 2010 (14)	64.2 ± 7.3	65.1 ± 6.9	98	95	rosiglitazone	Placebo treatment	12 months
Ahn et al. 2010 (15)	–	–	47	47	pioglitazone	Placebo treatment	8 months
Sung Hye You et al. 2010 (16)	59.4 ± 6.4	62.0 ± 7.4	19	18	pioglitazone	Placebo + basic treatment	8 months
Hideki Kitahara et al. 2011 (17)	66.4 ± 7.9	66.8 ± 9.6	25	25	pioglitazone	Placebo + basic treatment	9 months
Desch et al. 2010 (18)	61.3 ± 7.1	62.3 ± 6.5	16	12	rosiglitazone	Placebo treatment	6 months
Harald Sourij et al. 2006 (19)	–	–	21	21	pioglitazone	Placebo treatment	3 months
Yu et al. 2010 (20)	63.9 ± 2.2	63.3 ± 2.2	28	28	rosiglitazone	Basic treatment	3 months

I, Intervention group; C, control group; basic treatment, Placebo + aspirin 100 mg/d and clopidogrel 75 mg/d.

Our meta-analysis suggested that at baseline levels, there was no statistically significant difference between the intervention and control groups (all *P* > 0.05; Table 2).

**TABLE 2:** Baseline condition for the included experimental population.

Study ID	Age/Year		BMI		HbA1c (%)		FPG (mmol/L)	
	I	C	I	C	I	C	I	C
Marjorie Bastien et al. 2019 (13)	63.3 ± 7.4	64.8 ± 7.0	30.4 ± 3.8	28.7 ± 3.4	–	–	–	–
Olivier et al. 2010 (14)	64.2 ± 7.3	65.1 ± 6.9	30.2 ± 4.2	29.5 ± 4.6	6.9 ± 1.3	6.9 ± 0.8	7.7 ± 2.0	7.5 ± 1.6
Ahn et al. 2010 (15)	–	–	–	–	–	–	–	–
Sung Hye You et al. 2010 (16)	59.4 ± 6.4	62.0 ± 7.4	24.92 ± 2.04	26.05 ± 3.21	7.63 ± 1.40	7.73 ± 2.19	10.02 ± 4.18*	8.98 ± 2.07*
Hideki Kitahara et al. 2011 (17)	66.4 ± 7.9	66.8 ± 9.6	–	–	5.2 ± 0.3	5.2 ± 0.3	5.38 ± 0.33*	5.38 ± 0.38*
Desch et al. 2010 (18)	61.3 ± 7.1	62.3 ± 6.5	30.3 ± 3.9	31.3 ± 3.9	5.6 ± 0.2	5.8 ± 0.3	5.87 ± 0.48	6.08 ± 1.40
Harald Sourij et al. 2006 (19)	–	–	–	–	–	–	–	–
Yu et al. 2010 (20)	63.9 ± 2.2	63.3 ± 2.2	26.4 ± 0.6	24.7 ± 0.8	6.50 ± 0.26	6.78 ± 0.28	6.50 ± 0.34	6.43 ± 0.36

I, Intervention group; C, control group; *Conversion by the original document unit “mg/ml”.

### Risk of bias

**FIGURE 2:**
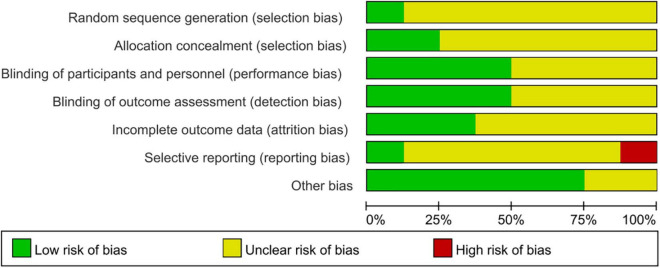
Schematic representation of the risk of bias.

All eight randomized controlled trials mentioned randomization and blindness methods. We evaluated the credibility of the information provided by the articles: 1 grouped by the randomized digital table; 1 literature mentioned single blindness, with the possibility of breaking blindness; the rest 6 mentioned blindness to researchers, subjects, and data analysts; insufficient data in the literature to indicate follow-up bias and reporting bias, no missing data and pre-reported outcomes have been reported. The results are shown in Figure 2.

### Thiazolidinediones delayed plaque progression

**FIGURE 3:**
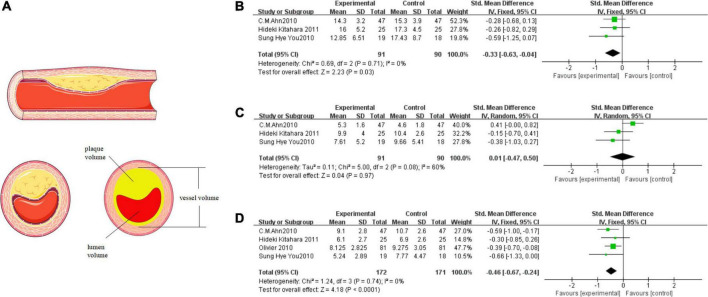
TZDs slowed the progression of plaque attachment on the vascular endothelium. **(A)** The relationship above vessel volume, lumen volume, and plaque volume; the materials in the figure were provided by Servier Medical Art (https://smart.servier.com) under a CC BY 3.0 license. **(B)** Effect of the TZDs on the vessel volume (mm^3^/mm). **(C)** Effect of the TZDs on the lumen volume (mm^3^/mm). **(D)** Effect of the TZDs on the plaque volume (mm^3^/mm).

We included three or four studies in the meta-analysis to evaluate the effect of TZDs on the plaque. As shown in Figure 3A, the values of plaque volume are the result of the subtraction of vessel volume and lumen volume. The data from three of the four publications are complete and the other one “Olivier 2020” records only the result of the subtraction (Figure 3D).

We found a low level of heterogeneity for vessel volume among the existing studies (*I*^2^ = 0%, *P* = 0.71) (Figure 3B). Based on the fixed-effects model of meta-analysis, lower levels of vessel volume were observed in the intervention group compared to the control subjects (SMD [95% CI]: −0.33 [−0.63, −0.04], *Z* = 2.23, *P* = 0.03). Then we found a medium level of heterogeneity for lumen volume among the existing studies (*I*^2^ = 60%, *P* = 0.08) (Figure 3C). Based on the random-effects model of meta-analysis the difference between the intervention group and the control group was not statistically significant (SMD [95% CI]:0.01 [−0.47, 0.50], *Z* = 0.04, *P* = 0.97). Heterogeneity mainly stems from the study “C. M. Ahn 2010”, which reported that the vessel volume decreased, the lumen volume increased, and the plaque volume decreased in the intervention group, and the final results were still in line with the overall trend. Finally, a very low level of heterogeneity for plaque volume was found among the existing studies (*I*^2^ = 0%, *P* = 0.74) (Figure 3D). Based on the fixed-effects model of meta-analysis, significantly lower levels of plaque volume were observed in the intervention group compared to the control subjects (SMD [95% CI]: −0.46 [−0.67, −0.24], *Z* = 4.18, *P* < 0.0001).

### Thiazolidinediones increased the adiponectin but didn’t affect the C reactive protein and interleukin-6 significantly

**FIGURE 4:**
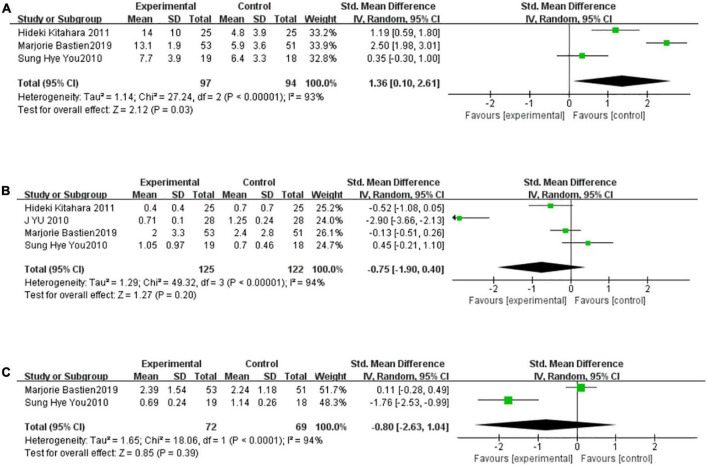
TZDs increased adiponectin but didn’t affect CRP and IL-6 significantly. **(A)** Effect of the TZDs on the adiponectin. **(B)** Effect of the TZDs on the CRP (mg/L). **(C)** Effect of the TZDs on the IL-6 (ng/L).

There were two to four studies to be included in the meta-analysis to evaluate the overall effect of TZDs on adiponectin, CRP, and IL-6. These indicators are thought to be closely related to inflammation. We found a high level of heterogeneity for adiponectin among the existing studies (*I^2^* = 93%, *P* < 0.00001) (Figure 4A). Based on the random-effects model of meta-analysis, significantly higher levels of adiponectin were observed in the intervention group compared to the control subjects. (SMD [95% CI]: 1.36[0.10, 2.61], *Z* = 2.12, *P* = 0.03). A high level of heterogeneity for CRP also appeared among the existing studies (*I*^2^ = 94%, *P* < 0.00001) (Figure 4B). Based on the random-effects model of meta-analysis, the difference was not statistically significant (SMD [95% CI]: −0.75[−1.90, 0.40], *Z* = 1.27, *P* = 0.20). Finally, only two studies were included in the meta-analysis to evaluate the overall effect of TZDs on IL-6. We found a high level of heterogeneity for IL-6 among the existing studies (*I*^2^ = 94%, *P* < 0.00001) (Figure 4C). Based on the random-effects model of meta-analysis, the difference was not statistically significant (SMD [95% CI]: −0.80[−2.63, 1.04], *Z* = 0.85, *P* = 0.39).

### Thiazolidinediones did not affect the serum levels of triglyceride, total cholesterol, low-density lipoprotein cholesterol, and high-density lipoprotein cholesterol

**FIGURE 5:**
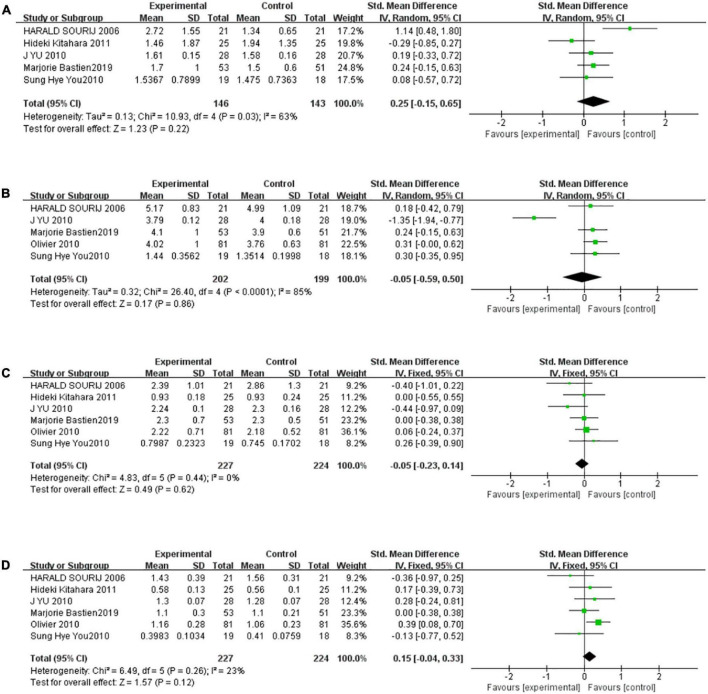
The effect of the TZDs on the serum lipid profile was unstable. **(A)** Effect of the TZDs on TG (mmol/L). **(B)** Effect of the TZDs on TC (mmol/L). **(C)** Effect of the TZDs on LDL-C (mmol/L). **(D)** Effect of the TZDs on HDL-C (mmol/L).

There were five studies to be included in the meta-analysis to evaluate the overall effect of TZDs on TG. We found a medium level of heterogeneity for TG among the existing studies (*I*^2^ = 63%, *P* = 0.03) (Figure 5A). Based on the random-effects model of meta-analysis, the difference was not statistically significant (SMD [95% CI]:0.25 [−0.15, 0.65], *Z* = 1.23, *P* = 0.22). For sensitivity analysis, the group heterogeneity was reduced by excluding the study “HARALD SOURIJ 2006” (*P* = 0.48, *I^2^* = 0%), higher levels of triglyceride were found in the intervention group compared to the control subjects in the fixed-effect model meta-analysis, but the difference was not statistically significant (SMD [95%CI]:0.10[−0.15,0.35], *Z* = 0.76, *P* = 0.44). Analogously, there were five studies to be included in the meta-analysis to evaluate the effect of TZDs on TC. We found a high level of heterogeneity for TC among the existing studies (*I*^2^ = 85%, *P* < 0.0001) (Figure 5B). Based on the random-effects model of meta-analysis, the difference was not statistically significant (SMD [95% CI]: −0.05 [−0.59, 0.50], *Z* = 0.17, *P* = 0.86). For sensitivity analysis, the group heterogeneity was reduced by excluding the study “J YU 2010”, (*P* = 0.98, *I*^2^ = 0%), and the fixed-effect model meta-analysis revealed that the intervention group’s total cholesterol levels were greater than those of the control subjects. (SMD [95% CI]:0.27 [0.06, 0.48], *Z* = 2.51, *P* = 0.01) In summary, the level of TC after the intervention of TZDs was unstable.

Six studies were included in the meta-analysis to evaluate the effect of TZDs on LDL-C. We found a very low level of heterogeneity for LDL-C among the existing studies (*I*^2^ = 0%, *P* = 0.44) (Figure 5C). Based on the fixed-effects model of meta-analysis, the difference was not statistically significant. (SMD [95% CI]: −0.05 [−0.23, 0.14], *Z* = 0.49, *P* = 0.62). Finally, there were six studies to be included in the meta-analysis to evaluate the overall effect of TZDs on HDL-C. We found a low level of heterogeneity for HDL-C among the existing studies (*I*^2^ = 23%, *P* = 0.26) (Figure 5D). Based on the fixed-effects model of meta-analysis, the difference was not statistically significant (SMD [95% CI]: 0.15 [−0.04, 0.33], *Z* = 1.57, *P* = 0.12).

## Discussion

According to previous reports, among the various types of medicine used in diabetes patients, TZDs were explored to have an inhibitory effect on cardiovascular disease by regulating serum lipid levels, serum adiponectin levels, and inflammatory response (21, 22). Research has shown that pioglitazone can reduce a range of cardiovascular risk factors, such as lipid unbalance, endothelial dysfunction, and inflammatory response (23). Furthermore, a randomized controlled trial with a large sample confirmed that TZDs have an anti-atherosclerotic effect (24). But there are still different opinions and controversies regarding the association between TZDs and the myocardial infarction to date. To demonstrate the role of TZDs in the treatment of diabetes combined with atherosclerosis, we performed this meta-analysis to explore the effect of TZDs on the plaque, vascular endothelium, and other indicators related to coronary atherosclerosis.

Based on our meta-analysis, in diabetic patients with coronary atherosclerosis, TZDs treatment resulted in an overall improvement in adiponectin and an inhibition of plaque volume. The results indicated that TZDs could protect the endothelium and reduce plaques. An essential function of the endothelium is to respond to physiological stimuli and produce transient vasodilators, including nitric oxide, bradykinin, and prostacyclin. The hyperglycemic state activates the endothelium and promotes foam macrophage formation. Those changes promote intimal thickening and promote endothelial dysfunction, then lead to the formation of plaque and atherosclerosis (25). TZDs treatment could attenuate atherosclerosis lesions partially by improving endothelial function, just as described in an article by Frank et al. (26).

Diabetes is a major risk factor for coronary heart disease (27), and the mortality rate of coronary heart disease is higher in diabetic patients than in non-diabetic subjects (28). Studies have shown that diabetic patients with concomitant coronary atherosclerosis have more extensive and severe coronary artery lesions (29), which may be associated with disorders of glucose metabolism, disorders of lipid metabolism, inflammatory response, and endothelial damage (30–32).

**FIGURE 6:**
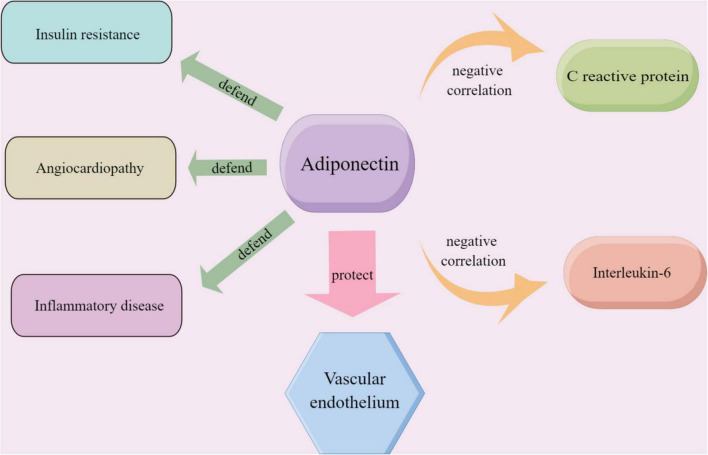
The role played by adiponectin in endothelium-related vascular diseases and the relationship between adiponectin and the inflammatory markers including CRP and IL-6; the material in the picture was provided Friendly by Figdraw (https://www.figdraw.com).

Inflammation and dyslipidemia are strongly associated with the formation of coronary atherosclerosis (33, 34). Adiponectin, an endocrine factor secreted by adipose tissue, plays an important role in insulin resistance, inflammation, and atherosclerosis in diabetes patients (35) (Figure 6). Adiponectin downregulates pro-inflammatory factors such as TNF-α and IL-6 and attenuates atherosclerosis by reducing inflammatory responses, lipid accumulation, and oxidative stress (36).

C reactive protein (CRP) is a sensitive marker of progressive systemic inflammation. And CRP and IL-6 are the most widely studied systemic markers of inflammation in cardiovascular disease. At the same time, adiponectin showed a significant negative correlation with CRP and IL-6 (37). In our study, the meta-analysis showed that serum adiponectin levels were elevated in the TZDs intervention group. It has been proved that TZDs activated the peroxisome proliferator-activated receptor isoforms (PPARγ), which alters the transcription of several genes involved in glycolipid metabolism (38) and thus affects adiponectin levels. On the one hand, clinical and experimental observations suggest that low serum adiponectin levels contribute to ameliorating insulin resistance in obese or overweight patients (39). On the other hand, low adiponectin expression is the cause of diabetic endothelial dysfunction (40).

For indicators of very high heterogeneity, we explored the source of the heterogeneity and removed it to test the stability of the results. It showed that lower levels of CRP and higher levels of TC were observed in the intervention group compared to the control subjects, but they are not entirely reliable (41). TZDs have been reported to affect lipid metabolism. In our study, we found that TZDs affected vessel plaque and adiponectin. And after TZDs treatment, those general inflammation indicators such as CRP and IL-6 only showed a trend of decline, rather than results with significant statistical differences. Unexpectedly, widely used indicators of blood lipids and inflammation, such as IL-6, LDL-C, and HDL-C didn’t exhibit statistical differences. We supposed these results may be due to the quantity and quality of the literature because there is a lot of strong evidence that inflammatory indices are closely related to adiponectin levels. An alternative explanation for blood lipid indicators is that the therapeutic effects of the TZDs maybe not depend on the regulation of lipid metabolism.

Our results suggested that TZDs ameliorated vascular endothelial and plaque-related indices in diabetic patients with atherosclerosis. Besides, our study showed that TZDs didn’t affect serum lipid indicators, indicating that the above effects of TZDs may not depend on the status of serum lipids. Since the endothelium and plaque are very closely related to myocardial infarction, we can then suppose that TZDs may eventually play a protective role in myocardial infarction by raising adiponectin, protecting the endothelium, and delaying plaque development. Recently, in the treatment of diabetes, there have emerged drugs thought to have cardiovascular benefits, such as sodium-glucose cotransporter 2 inhibitors (SGLT-2i) and glucagon-like peptide-1 receptor agonists (GLP-1RAs), these drugs exert glucose-lowering effects through novel therapeutic targets, and may play positive roles in coronary atherosclerosis. It has been reported that SGLT2-i treatment accelerated features of plaque stability during atherosclerosis regression (42), and GLP-1RAs alleviated vascular inflammation and plaque burden (43). Both of the drugs have positive effects on the treatment of plaque, then exert potential therapeutic effects on coronary atherosclerosis, which is worth further discussion. Those above provide new ideas for ensuring optimal treatment with few adverse events and unnecessary multiple drugs combination.

Some limitations in our paper: low levels of the literature included; high heterogeneity between studies, and rough integration of rosiglitazone with pioglitazone creates bias. We look forward to more high-quality researches in this field. If the sample size is larger, our conclusion that “TZDs ameliorated the vascular endothelium and plaque in diabetic with atherosclerosis and developed a potential protective effect on myocardial infarction which did not depend on the improvement of lipid profile” may be more reasonable.

## Data availability statement

The original contributions presented in this study are included in the article/Supplementary material, further inquiries can be directed to the corresponding authors.

## Author contributions

CYX mainly performed the data collection, with assistance from MQZ. QYZ performed the data analysis and interpretation. CYX and LXL drafted the manuscript. WJL provided the critical revisions. All authors contributed to developing the study concept, designing the study, and approved the final version of the manuscript for submission.

## Footnotes

1
https://training.cochrane.org/online-learning/core-software-cochrane-reviews/revman
