# Supplementary material for: Thiazolidinediones play a positive role in the vascular endothelium and inhibit plaque progression in diabetic patients with coronary atherosclerosis: A systematic review and meta-analysis
Source: Front Cardiovasc Med. 2022 Nov 29;9:1043406. doi: 10.3389/fcvm.2022.1043406 (PMC9744793; doi:10.3389/fcvm.2022.1043406)
Supplement: Supplementary file 1 [file Data_Sheet_1.docx]

Supplementary Material

# Table S1——The retrieval formula for the Meta-analysis

**The PubMed retrieval formula**

((("Endothelium"[MeSH Terms] OR "Endotheliums"[Title/Abstract]) AND "thiazolidinediones"[MeSH Terms]) OR "Glitazones"[Title/Abstract] OR "Rosiglitazone"[MeSH Terms] OR "rosiglitazone maleate"[Title/Abstract] OR "Avandia"[Title/Abstract] OR "Pioglitazone"[MeSH Terms] OR ("pioglitazone hydrochloride"[Title/Abstract] OR "Actos"[Title/Abstract])) AND ("Coronary Disease"[MeSH Terms] OR "coronary atherosclerosis"[Title/Abstract] OR "Artery Diseases,Coronary"[Title/Abstract] OR "Coronary Artery Diseases"[Title/Abstract] OR "Coronary Arteriosclerosis"[Title/Abstract] OR "Atherosclerosis,Coronary"[Title/Abstract] OR "Arteriosclerosis,Coronary"[Title/Abstract] OR "Disease,Coronary"[Title/Abstract] OR "Diseases,Coronary"[Title/Abstract] OR "Coronary Heart Disease"[Title/Abstract] OR "Coronary Heart Diseases"[Title/Abstract] OR "Disease,Coronary Heart"[Title/Abstract] OR "Diseases,Coronary Heart"[Title/Abstract] OR "Heart Disease,Coronary"[Title/Abstract] OR "Heart Diseases,Coronary"[Title/Abstract]) AND ("randomized controlled trial"[Publication Type] OR "randomized"[Title/Abstract])

**The Embase retrieval formula**

No. Query Results

#31. #20 AND #26 AND #29 AND #30 10

#30. 'random':ti,ab OR 'placebo':ti,ab OR 741,724

'double-blind':ti,ab

#29. #27 OR #28 143,824

#28. endotheliums:ti,ab 16

#27. 'endothelium'/exp 143,821

#26. #21 OR #22 OR #23 OR #24 OR #25 45,572

#25. 'actos':ti,ab 134

#24. 'pioglitazone hydrochloride':ti,ab 231

#23. 'avandia':ti,ab 164

#22. 'rosiglitazone maleate':ti,ab 138

#21. 'glitazone derivative'/exp OR '2,4 45,538

thiazolidinedione derivative'/exp OR

'rosiglitazone'/exp OR 'pioglitazone'/exp

#20. #1 OR #2 OR #3 OR #4 OR #5 OR #6 OR #7 OR #8 OR 749,460

#9 OR #10 OR #11 OR #12 OR #13 OR #14 OR #15 OR

#16 OR #17 OR #18 OR #19

#19. 'heart diseases, coronary':ti,ab 38

#18. 'heart disease, coronary':ti,ab 389

#17. 'diseases, coronary heart':ti,ab 137

#16. 'disease, coronary heart':ti,ab 421

#15. 'coronary heart diseases':ti,ab 1,534

#14. 'coronary heart disease':ti,ab 69,692

#13. 'diseases, coronary':ti,ab 333

#12. 'disease, coronary':ti,ab 2,197

#11. 'arteriosclerosis, coronary':ti,ab 41

#10. 'coronary atheroscleroses':ti,ab 2

#9. 'atheroscleroses, coronary':ti,ab 0

#8. 'atherosclerosis, coronary':ti,ab 575

#7. 'coronary arterioscleroses':ti,ab 0

#6. 'arterioscleroses, coronary':ti,ab 0

#5. 'coronary arteriosclerosis':ti,ab 818

#4. 'coronary artery diseases':ti,ab 2,180

#3. 'artery diseases, coronary':ti,ab 2

#2. 'coronary atherosclerosis':ti,ab 11,834

#1. 'coronary artery atherosclerosis'/exp OR 724,208

'ischemic heart disease'/exp

**The Cochrane library retrieval formula**

ID Search Hits

#1 MeSH descriptor: [Myocardial Ischemia] explode all trees 28926

#2 (coronary atherosclerosis):ti,ab,kw OR (artery diseases, coronary):ti,ab,kw OR (coronary artery diseases):ti,ab,kw OR (coronary arteriosclerosis):ti,ab,kw OR (atherosclerosis, coronary):ti,ab,kw 6861

#3 (arteriosclerosis, coronary):ti,ab,kw OR (disease, coronary):ti,ab,kw OR (diseases, coronary):ti,ab,kw OR (coronary heart disease):ti,ab,kw OR (coronary heart diseases):ti,ab,kw 37203

#4 (disease, coronary heart):ti,ab,kw OR (diseases, coronary heart):ti,ab,kw OR (heart disease, coronary):ti,ab,kw OR (heart diseases, coronary):ti,ab,kw 22045

#5 (#1 or #2 or #3 or #4) 50720

#6 (Glitazones):ti,ab,kw 62

#7 MeSH descriptor: [Rosiglitazone] explode all trees 627

#8 MeSH descriptor: [Thiazolidinediones] explode all trees 1839

#9 (Rosiglitazone Maleate):ti,ab,kw OR (Avandia):ti,ab,kw 67

#10 MeSH descriptor: [Pioglitazone] explode all trees 1075

#11 (Pioglitazone Hydrochloride):ti,ab,kw OR (Actos):ti,ab,kw 1231

#12 (#6 or #7 or #8 or #9 or #10 or #11) 3061

#13 MeSH descriptor: [Endothelium] explode all trees 3190

#14 (#5 and #12 and #13) 15

**The Web of Science retrieval formula**

#1 TS=(Endothelium OR Endotheliums )

#2 TS=(Thiazolidinediones OR Glitazones OR Rosiglitazone OR Rosiglitazone Maleate OR Avandia OR Pioglitazone OR Pioglitazone Hydrochloride OR Actos)

#3 TS=(Coronary Disease OR coronary atherosclerosis OR Artery Diseases,Coronary OR Coronary Artery Diseases OR Coronary Arteriosclerosis OR Atherosclerosis,Coronary OR Arteriosclerosis,Coronary OR Disease,Coronary OR Diseases,Coronary OR Coronary Heart Disease OR Coronary Heart Diseases OR Disease,Coronary Heart OR Diseases,Coronary Heart OR Heart Disease,Coronary )

#4 TS=(random* controlled trial OR random* OR placebo)

#5 #1 AND #2 AND #3 AND #4
